# Clinicopathological significance of SOX4 expression in primary gallbladder carcinoma

**DOI:** 10.1186/1746-1596-7-41

**Published:** 2012-04-17

**Authors:** Chengguo Wang, Huadong Zhao, Jianguo Lu, Jikai Yin, Li Zang, Nuan Song, Rui Dong, Tao Wu, Xilin Du

**Affiliations:** 1 Department of general surgery, Tangdu Hospital, Fourth Military Medical University, Xi'an 710038, People's Republic of China

**Keywords:** Primary gallbladder carcinoma, SOX4, Clinicopathology, Overall survival, Disease-free survival

## Abstract

**Aim:**

SOX4, as a member of the SRY-related HMG-box (SOX) transcription factor family, has been demonstrated to be involved in tumorigenesis of many human malignancies; however, its role in primary gallbladder carcinoma (PGC) is still largely unknown. The aim of this study was to investigate SOX4 expression in PGC and its prognostic significance.

**Methods:**

From 1997 to 2006, 136 patients underwent resection for PGC. The median follow-up was 12.8 months. Immunostainings for SOX4 were performed on these archival tissues. The correlation of SOX4 expression with clinicopathological features including survival was analyzed.

**Results:**

SOX4 was expressed in 75.0% (102/136) of PGC but not in the normal epithelium of the gallbladder. In addition, the over-expression of SOX4 was significantly associated with low histologic grade (*P *= 0.02), low pathologic T stage (*P *= 0.02), and early clinical stage (*P *= 0.03). The levels of SOX4 immunostainings in PGC tissues with positive nodal metastasis were also significantly lower than those without (*P *= 0.01). Moreover, Kaplan-Meier curves showed that SOX4 over-expression was significantly related to better overall (*P *= 0.008) and disease-free survival (*P *= 0.01). Furthermore, multivariate analyses showed that SOX4 expression was an independent risk factor for both overall (*P *= 0.03, hazard ratio, 3.682) and disease-free survival (*P *= 0.04, hazard ratio, 2.215).

**Conclusion:**

Our data indicate for the first time that the over-expression of SOX4 in PGC was significantly correlated with favorable clinicopathologic features and was an independent prognostic factor for better overall and disease-free survival in patients. Therefore, SOX4 might be an auxiliary parameter for predicting malignant behavior for PGC.

**Virtual slides:**

The virtual slide(s) for this article can be found here: http://www.diagnosticpathology.diagnomx.eu/vs/1534825818694957.

## Introduction

Primary gallbladder carcinoma (PGC) is one of the most common malignancies of the digestive tract in China. In the last two decades, the diagnosis and therapeutic technologies have been greatly improved; however, the clinical outcome of patients with PGC remains poor, because of the early spread of tumors by lymphatic, perineural and hematogenous routes and direct invasion into the liver. There is no specific symptom for PGC patients. So the diagnosis of this carcinoma is usually made postoperatively on tumors at an advanced stage; almost half of patients already have metastatic disease at the time of surgery [1]. Similar with other various human malignancies, multiple genetic or epigenetic changes also contribute to the multistep process of PGC, and some of these changes may help monitor this multistep process [2]. Therefore, it is necessary to understand the carcinogenic process and its corresponding molecular basis for PGC, which may provide a useful insight that aid in the evaluation of prognostic factors, the establishment of new therapeutic strategies, and the improvement of patients' survival.

The SOX (sex-determining region Y-related high mobility group [HMG] box) transcription factor family plays a key role in many aspects of development, including sex determination, testis formation, neuronal development, lymphocyte differentiation and chondrogenesis [3]. Members in this family share the highly conserved HMG box, which mediates binding of SOX proteins to a short-target DNA sequence directly [4]. In vertebrates, there have been more than 20 genes identified as members of SOX family, and they have been categorized into groups A-G according to their sequence similarity. SOX4, one of group-C SOX genes, has been shown to be involved in a range of developmental processes, such as embryonic cardiac development, nervous system development, osteoblastic differentiation, and thymocyte development [5]. The SOX4 gene encodes a protein of 474 amino acids with three distinguishable domains: an HMG box, a glycine-rich region and a serine-rich region. The HMG box serves as a DNA-binding region, whereas the serine-rich domain serves as a transactivation domain [6]. The central domain containing the glycine-rich region located between the HMG box and serine-rich domains serves as a novel functional region for promoting apoptotic cell death [7]. Recently, it has been demonstrated that SOX4 is involved in tumorigenesis of many human malignancies. The up-regulation of SOX4 has been detected in breast cancer, pancreatic cancer, lung cancer, prostate cancer, colon cancer, meduloblastoma, ovarian cancer and hepatocellular carcinoma [8-12]. In addition, Aaboe et al. [13] found that the strong SOX4 expression was correlated with increased survival of patients with bladder cancer, and it also impaired tumor cell viability and promoted apoptosis. Hur et al. [14] reported that SOX4 contributes to hepatocarcinogenesis by inhibiting p53-mediated apoptosis and that its overexpression might be a useful prognostic marker for better survival in patients with hepatocellular carcinoma after surgical resection. However, its role in PGC is still largely unknown. To address this problem, the aim of this study was to investigate SOX4 expression in PGC and its prognostic significance.

## Materials and methods

### Patients and tissue samples

The study was approved by the Research Ethics Committee of Department of general surgery, Tangdu Hospital, Fourth Military Medical University, Xi'an, P.R. China. Informed consent was obtained from all of the patients. All specimens were handled and made anonymous according to the ethical and legal standards.

Prospectively collected data of 136 patients (60 men and 76 women), who underwent surgery for PGC between November 1997 and November 2006, were reviewed. The mean age of the patients was 66 years (range, 30-87 years). A curative resection (R0) was defined as negative resection margins by light microscopical examination. For each patient prospectively registered clinicopathological variables were extracted from the electronic clinical records: demographic data (age, gender), presenting symptoms, biochemistry, and surgical therapy. All the pathology slides were reviewed by two pathologists with special attention for tumor growth pattern and differentiation, the pathologic margin status, the presence of lymphovascular invasion, perineural invasion and the total number and status of regional and distant lymph nodes harvested. Tumor stage was classified according to the American Joint Committee on Cancer system. For histologic grading, the PGC specimens were examined by routine hematoxylin and eosin staining. The specimens were graded into well (G1), moderately (G2), poorly differentiated (G3), and undifferentiated (G4) adenocarcinoma according to the World Health Organization classification. The clinicopathological features of the patients are summarized in Table 1.

**Table 1:** Association of SOX4 expression with the clinicopathological features of 136 patients with PGC

Factor	**NO**.	SOX4 expression (n, %)				*P*
		
		Negative	Weak	Moderate	Strong	
**Gender**						
Male	60	14 (23.3)	9 (15.0)	20 (33.3)	17 (28.3)	NS
Female	76	20 (22.2)	12 (15.8)	25 (32.9)	19 (25.0)	
**Age**						
≤ 66 years	65	16 (24.6)	10 (15.4)	21 (32.3)	18 (27.7)	NS
> 66 years	71	18 (25.4)	11 (15.5)	24 (33.8)	18 (25.4)	
**Tumor size**						
≤ 2.5 cm	72	19 (26.4)	12 (16.7)	25 (34.7)	16 (22.2)	NS
> 2.5 cm	64	15 (23.4)	9 (14.1)	20 (31.3)	20 (31.3)	
**Histological grade**						
G1	18	1 (5.6)	3 (16.7)	4 (22.2)	10 (55.6)	**0.02**
G2	72	13 (18.1)	3 (4.2)	32 (44.4)	24 (33.3)	
G3	33	10 (30.3)	13 (39.4)	8 (24.2)	2 (6.1)	
G4	13	10 (76.9)	2 (15.4)	1 (7.7)	0 (0.0)	
**Pathologic T stage**						
T1	22	2 (9.1)	2 (9.1)	6 (27.3)	12 (54.5)	**0.02**
T2	60	4 (6.7)	5 (8.3)	29 (48.3)	22 (36.7)	
T3	36	16 (44.4)	10 (27.8)	8 (22.2)	2 (5.6)	
T4	18	12 (66.7)	4 (22.2)	2 (11.1)	0 (0.0)	
**Clinical stage**						
I	46	4 (8.7)	6 (13.0)	16 (2.2)	20 (43.5)	**0.03**
II	52	8 (15.4)	4 (7.7)	25 (48.1)	15 (28.8)	
III	18	10 (55.6)	5 (27.8)	2 (11.1)	1 (5.6)	
IV	20	12(60.0)	6 (30.0)	2 (10.0)	0 (0.0)	
**Nodal metastasis**						
Negative	92	12 (13.0)	3 (3.3)	41 (44.6)	36 (39.1)	**0.01**
Positive	44	22 (50.0)	18 (40.9)	4 (9.1)	0 (0.0)	
**Distant metastasis**						
Negative	112	28 (25.0)	16 (14.3)	35 (31.3)	33 (29.5)	NS
Positive	24	6 (25.0)	5 (20.8)	10 (41.7)	3 (12.5)	
**Venous/lymphatic invasion**						
Negative	82	22 (26.8)	12 (14.6)	24 (29.3)	24 (29.3)	NS
Positive	54	12 (22.2)	9 (16.7)	21 (38.9)	12 (22.2)	
**Perineural invasion**						
Negative	79	20 (25.3)	12 (15.2)	24 (30.4)	23 (29.1)	NS
Positive	57	14 (24.6)	9 (15.8)	21 (36.8)	13 (22.8)	

**Note: **'NS' refers to 'No statistic significance'

Follow-up data were recorded from the patient's medical records and completed by a telephone survey performed on July 2011. Overall survival (OS) was defined as the time (months) from the date of surgery to the date of death by PGC. Disease-free survival (DFS) was defined as the time (months) from the date of surgery to the date of the first recurrence confirmed by imaging modalities. Median post-operative follow-up was 12.8 months (range: 22 days ~ 126.3 months). During the follow-up period, thirty-two patients (23.5%) were still alive, but 104 patients (76.5%) died. The overall mean ± SEM survival time of the 136 patients was 29.6 ± 1.8 months. There was no perioperative mortality.

### Immunohistochemical staining and assessment

For immunohistochemical staining, formalin-fixed paraffin-embedded tissue was cut into 4-μm sections. Commercially available mouse-antihuman monoclonal antibody against SOX4 (1:100 dilution; American Research Products, Belmont, Mass) was used. The specificity of the primary antibody against SOX4 has been demonstrated by the previous study of Shen et al. [15]. Immunohistochemical staining was carried out on sections using the avidin-biotin method and a commercially available kit (Vectastain Elite ABC kit, Vector Laboratories, Burlingame, CA). Deparaffinized sections were treated with methanol containing 3% hydrogen peroxide for 10 min before conducting antigen retrieval using a microwave oven at 95°C for 5 min and cooling at 25°C for 2 h. After washing with PBS, blocking serum was applied for 10 min. The sections were incubated with anti-SOX4 monoclonal antibody overnight at 4°C. After washing in PBS, a biotin-marked goat anti-mouse secondary antibody was applied for 10 min followed by a peroxidase-marked streptavidin for an additional 10 min. The reaction was visualized by using 3, 3'-diaminobenzidine tetrahydrochloride. The nuclei were counterstained with hematoxylin. Positive and negative immunohistochemistry controls were routinely used. Reproducibility of staining was confirmed by reimmunostaining via the same method in multiple, randomly selected specimens.

Immunoreactivity was assessed by two investigators who were blinded to clinicopathologic data. Discrepancies were resolved by simultaneous reexamination of the slides by both investigators using a double-headed microscope. Scoring of immunohistochemistry was based on two parameters: the proportion of immunopositive cells and their intensity of immunoreactivity. The proportion of immunopositive cells was categorized as follows: 0: < 10%; 1: ≥ 10% to < 25%; 2: ≥ 25% to < 50%; 3: ≥ 50% to < 75% and 4: ≥ 75%. The staining intensity was categorized by relative intensity as follows: 0: no positivity; 1: weak; 2: moderate and 3: strong. A final immunoreactivity score of each section was obtained by multiplying the two individual scores and was divided into four levels: '0', negative; '1 ~ 4', weak; '5 ~ 8', moderate; '9 ~ 12', strong.

### Statistical analysis

SPSS13.0 software for Windows (SPSS Inc, USA) was used for statistical analysis. Continuous variables were expressed as $\bar{X}\pm s$. Group comparisons of categorical variables were evaluated using the Fisher's exact or Pearson's chi-square test. Kaplan-Meier method was used for the question of survival. Chiquest trend test and Cox regression analysis were performed for ordinal datum and the multivariate analysis, respectively. The *p *values of less than 0.05 were considered to be statistically significant.

## Results

### Immunohistochemical findings of SOX4 in PGC

**Figure 1:**
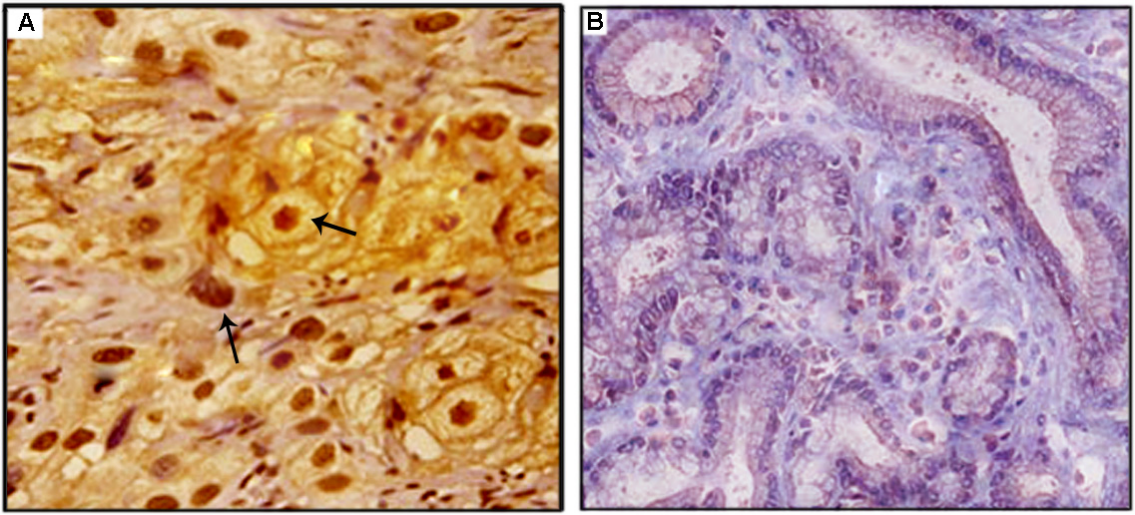
**Immunohistochemical staining of SOX4 expression in PGC**. Commercially available mouse-antihuman monoclonal antibody against SOX4 was used to stain the paraffin section. A, Strong cytoplasmic and nuclear expression of SOX4 was detected at the tumor cells of PGC tissues (original magnification × 400); B, SOX4 was negative in normal epithelium of gallbladder (original magnification × 400).

To assess the biological significance of SOX4 expression in gallbladder tumors, we evaluated its expression in 136 PGC samples from PGC patients with full clinical annotation. As the results, the expression of SOX4 was observed in both cell nucleus and cytoplasm of tumor cells in PGC samples (Figure 1A) which was consistent with previous studies [11,12], and varied in intensity and extent of staining in different tumors. SOX4 expression was not detected in the normal epithelium of the gallbladder (Figure 1B). In addition, SOX4 was expressed in 75.0% (102/136) tumor samples and was strongly expressed in 36 (26.5%), moderately expressed in 45 (33.1%), and weakly expressed in 21 (15.4%), and not expressed in 34 (25.0%) tumor samples.

### Association between SOX4 expression and the clinicopathological features of PGC

Various clinicopathological features of PGC patients and their tumors were compared based on the expression levels of SOX4 they had. The analysis revealed that the tumors with strong SOX4 expression less frequently showed positive nodal metastasis (*P *= 0.01). The tumors with the over-expression of SOX4 tended to show low histologic grade (*P *= 0.02), low pathologic T stage (*P *= 0.02), and early clinical stage (*P *= 0.03). There was no significant association with age, gender, or tumor size (Table 1).

### Prognostic value of SOX4 expression in PGC

**Figure 2:**
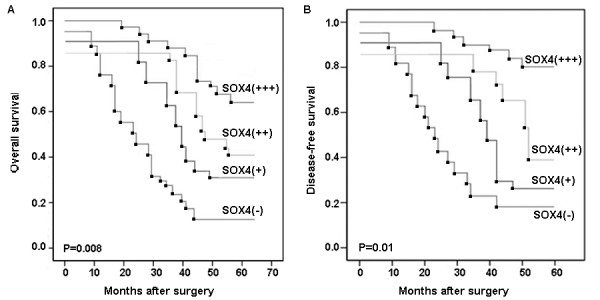
**Overall survival (A) and disease-free survival (B) in 136 patients with PGC according to the expression levels of SOX4**. The analysis with Kaplan-Meier method clearly showed that PGC patients having tumors with strong SOX4 expression (+++) had respectively increased overall (*P *= 0.008) and DFS (*P *= 0.01) compared with patients with negative (-), weak (+) or moderate (++) SOX4 expression.

Adequate clinical follow-up information was available for all 136 cases. We investigated the prognostic value of SOX4 expression in PGC. The OS rates of SOX4 negative and SOX4 positive (weak ~ strong) were 11.8% and 45.1%, respectively (*P *< 0.001), and the DFS rate of SOX4 negative and SOX4 positive (weak ~ strong) was 14.7% and 49.0%, respectively (*P *< 0.001). The survival curves according to SOX4 expression are shown in Figure 2. The analysis with Kaplan-Meier method clearly showed that PGC patients having tumors with strong SOX4 expression had respectively increased OS and DFS compared with patients with negative, weak or moderate SOX4 expression (*P *= 0.008 and 0.01, Figure 2A and 2B, respectively).

Then, we estimated the clinical significance of various prognostic factors that might influence survival and recurrence. As summarized in Table 2, the univariate analysis suggested that advanced pathologic T stage (*P *= 0.008), advanced clinical stage (*P *< 0.001), nodal metastasis (*P *= 0.001), distant metastasis (*P *= 0.001), and negative SOX4 expression (*P *= 0.01) were statistically significant risk factors affecting OS of patients with PGC. In case of DFS, advanced pathologic T stage (*P *= 0.02), advanced clinical stage (*P *= 0.001), nodal metastasis (*P *= 0.001), and negative SOX4 expression (*P *= 0.02) were statistically significant risk factors.

**Table 2:** Univariate analysis of the association between prognosis and various clinicopathologic parameters in patients with PGC

**Features**	Overall survival			Disease-free survival		
	Hazard ratio	95% CI	P	Hazard ratio	95% CI	P
**Gender (Male vs. Female)**	0.672	0.328-1.296	NS	1.007	0.426-2.335	NS
**Age (≤ 66 years vs**.**≥ 66 years)**	0.766	0.357-1.402	NS	0.890	0.368-1.563	NS
**Tumor size (≤ 2.5 cm vs**.**≥ 2.5 cm)**	0.819	0.334-1.628	NS	1.023	0.269-2.682	NS
**Histological grade (G1-G2 vs. G3****± G4)**	0.981	0.365-2.879	NS	1.107	0.319-2.989	NS
**Pathologic T stage (T1****± T2 vs. T3 ± T4)**	3.387	1.528-8.336	0.008	3.072	1.269-8.668	0.02
**Clinical stage (I****± II vs. III ± IV)**	5.620	1.591-11.072	< 0.001	4.863	1.893-10.651	0.001
**Nodal metastasis (Negative vs. Positive)**	4.701	1.310-8.543	0.001	4.663	1.700-10.253	0.001
**Distant metastasis (Negative vs. Positive)**	4.509	1.271-9.775	0.001	2.181	1.037-4.357	NS
**Venous/lymphatic invasion (Negative vs. Positive)**	2.687	1.190-5.053	NS	2.508	1.431-4.383	NS
**Perineural invasion (Negative vs. Positive)**	1.216	1.087-3.126	NS	1.246	1.507-3.608	NS
**SOX4 expression (Negative vs. Positive)**	3.237	1.221-7.952	0.01	3.782	1.339-8.862	0.02

**Note: **'NS' refers to 'No statistic significance'

Furthermore, we evaluated the independent prognostic impacts of these various factors. As summarized in Table 3, the multivariate analysis using the Cox proportional hazards model shown that advanced clinical stage (hazard ratio [HR], 8.862; 95% confidence interval [CI], 1.328-26.523; *P *= 0.008) and negative SOX4 expression (HR, 3.682; 95% CI, 1.102-10.282; *P *= 0.03) were independent risk factors predicting short OS. In case of DFS, advanced clinical stage (HR, 3.391; 95% CI, 1.183-9.923; *P *= 0.02) and negative SOX4 expression (HR, 2.215; 95%CI, 1.088-7.016; *P *= 0.04) were independent risk factors predicting short DFS (Table 3).

**Table 3:** Multivariate analysis of the association between prognosis and various clinicopathologic parameters in patients with PGC

Features	Overall survival			Disease-free survival		
	
	Hazard ratio	95% CI	P	Hazard ratio	95% CI	P
**Pathologic T stage (T1****± T2 vs. T3 ± T4)**	0.528	0.106-5.291	NS	1.066	0.281-6.893	NS
**Clinical stage (I****± II vs. III ± IV)**	8.862	1.328-26.523	0.008	3.391	1.183-9.923	0.02
**Nodal metastasis (Negative vs. Positive)**	1.728	0.553-6.965	NS	1.625	0.685-7.097	NS
**Distant metastasis (Negative vs. Positive)**	1.763	1.128-6.553	NS	1.711	0.737-7.267	NS
**Venous/lymphatic invasion (Negative vs. Positive)**	2.181	1.018-8.106	NS	2.527	1.023-8.298	NS
**Perineural invasion (Negative vs. Positive)**	0.826	0.115-3.089	NS	0.908	0.259-3.765	NS
**SOX4 expression (Negative vs. Positive)**	3.682	1.102-10.282	0.03	2.215	1.088-7.016	0.04

**Note: **'NS' refers to 'No statistic significance'

## Discussion

PGC is an aggressive and lethal cancer. Patients with this carcinoma are usually treated at an advanced stage, and the prognosis remains poor despite the development of modern diagnostic methods. Therefore, it is necessary to understand the mechanisms responsible for the characteristic growth and metastasis of PGC. In the present study, the immunohistochemical expression of the SOX4 protein in a large number of PGC was analyzed. There are four points of our findings. Firstly, the SOX4 protein was up-regulated in tumor cells of PGC tissues, but not expressed in normal epithelium of gallbladder; Secondly, the decreased SOX4 expression in PGC tissues was significantly correlated with advanced tumor progression and aggressive clinicopathological features; Thirdly, the results of Kaplan-Meier analyses shown that PGC tissues with strong SOX4 expression tend to have increased OS and DFS rates respectively. Finally, both univariate and multivariate analyses clearly demonstrated that the negative SOX4 expression was a statistically significant risk factor affecting OS and DFS of patients with PGC, suggesting that SOX4 expression could be a useful marker to predict patient survival.

The SOX4 gene, localized to 6p22.3, is highly conserved in vertebrates [16]. It has 88% identity at the DNA level between Homo sapiens and Fugu rubripes in the NH_2_-terminal domain [17]. The SOX4 gene encodes a 47 kDa protein of the SOX family, which may play roles in the development of cancer cells. For one thing, SOX4 has been demonstrated as a tumor suppressor gene. For example, Aaboe et al. [13] in 2006 reported that the overexpression of SOX4 in bladder carcinoma cell lines could strongly impaired cell viability and promoted apoptosis. They also found a correlation between strong SOX4 expression and increased survival in patients with bladder carcinoma. Pan et al. [18] in 2009 found that SOX4 as a new DNA damage sensor is required for the activation of p53 tumor suppressor in response to DNA damage. It interacts with and stabilizes p53 protein by blocking Mdm2-mediated p53 ubiquitination and degradation, and also enhances p53 acetylation by interacting with p300/CBP and facilitating p300/CBP/p53 complex formation. Consequently, SOX4 promotes cell cycle arrest and apoptosis, and it inhibits tumorigenesis in a p53-dependent manner. In 2010, Hur et al. [14] further demonstrated that SOX4 interacts with p53 using its HMG box domain, which leads to the inhibition of p53-mediated transcription by the Bax promoter. In clinicopathological analysis, they also indicated that the nuclear overexpression of SOX4 was observed in tumor cells of hepatocellular carcinoma tissues, which was correlated with diminished risk of recurrence and improved overall survival time in patients. For the other thing, SOX4 also acts as an oncogene in some tumors. For example, Chen et al. [19] in 2007 found that SOX4 gene mutation is significantly associated with pathological stages and the mutation rate increases gradually, which has relation with advanced pathological stages in non-small cell lung cancer tissues, suggesting that the SOX4 gene mutations might be related in the lung carcinogenesis and tumor metastasis. In 2009, Medina et al. [20] emphasize the oncogenic properties of SOX4 and show the interaction between gene amplification at 6p and SOX4 overexpression in lung cancer. Therefore, whether SOX4 may act as a tumor suppressor or an oncogene seems to be determined by the function of its interactors and the signal transduction it is involved in. Consistent with the findings of Aaboe et al. [13] in bladder carcinoma and Hur et al. [14] in hepatocellular carcinoma, our data shown that the expression levels of SOX4 in moderately or poorly differentiated PGC was significantly lower than that in well-differentiated PGC. The decreased expression of SOX4 was also significantly associated with advanced pathologic T stage, advanced clinical stage, and positive nodal metastasis, suggesting that SOX4 expression might be of clinical relevance in the aggressiveness of PGC. We further demonstrated that the SOX4 expression was associated with a favorable outcome in patients with PGC.

In conclusion, our data have provided evidence for the first time that the SOX4 protein is highly up-regulated in tumor cells of PGC tissues. We have shown that strong SOX4 protein expression is correlated with less agressive clinicopathological features and increased patient survival. Therefore, SOX4 might be an auxiliary parameter for predicting malignant behavior for PGC. However, the precise mechanism that SOX4 is involved in the tumorigensis and tumor progression of PGC remains unclear. The further prospective analysis would be worth doing.

## Competing interests

The authors declare that they have no competing interests.

## Authors' contributions

WCG, ZHD, WT and DXL: participated in study design and coordination, analysis and interpretation of data, material support for obtained funding, and supervised study; LJG, YJK, and ZL: help to translated and edit the paper; SN and DR: carry out part of the experiments. All authors read and approved the final manuscript.
